# Levosimendan vs. Dobutamine in Patients with Septic Shock: A Systematic Review and Meta-Analysis with Trial Sequential Analysis

**DOI:** 10.3390/jcm14155496

**Published:** 2025-08-05

**Authors:** Edith Elianna Rodríguez, German Alberto Devia Jaramillo, Lissa María Rivera Cuellar, Santiago Eduardo Pérez Herran, David René Rodríguez Lima, Antoine Herpain

**Affiliations:** 1 Critical and Intensive Care Medicine, Hospital Universitario Mayor-Méderi, Bogotá 111411, Colombia; davidre.rodriguez@urosario.edu.co; 2 Emergency Medicine, Fundación Santa Fe de Bogotá, Bogotá 110111, Colombia; german.devia@urosario.edu.co; 3 Escuela de Medicina y Ciencias de la Salud, Universidad del Rosario, Bogotá 111221, Colombia; lissa.rivera@urosario.edu.co (L.M.R.C.); santiagoe.perez@urosario.edu.co (S.E.P.H.); 4 Grupo de Investigación Clínica, Escuela de Medicina y Ciencias de la Salud, Universidad del Rosario, Bogotá 111221, Colombia; 5 Department of Intensive Care, Hôpital Erasme, 1070 Brussels, Belgium; antoine.herpain@ulb.be

**Keywords:** levosimendan, dobutamine, septic shock, myocardial disfunction

## Abstract

**Introduction:** Septic-induced cardiomyopathy (SICM) is a life-threatening condition in patients with septic shock. Persistent hypoperfusion despite adequate volume status and vasopressor use is associated with poor outcomes and is currently managed with inotropes. However, the superiority of available inotropic agents remains unclear. This meta-analysis aims to determine which inotropic agent may be more effective in this clinical scenario. **Methods:** A systematic review and meta-analysis were conducted, including data from randomized clinical trials (RCTs) comparing levosimendan and dobutamine in patients with septic shock and persistent hypoperfusion. Summary effect estimates, including odds ratios (ORs), standardized mean differences (SMDs), and 95% confidence intervals (CIs), were calculated using a random-effects model. Trial sequential analysis (TSA) was also performed. **Results:** Of 244 studies screened, 11 RCTs were included. Levosimendan was associated with a reduction in in-hospital mortality (OR 0.64; 95% CI: 0.47; 0.88) and ICU length of stay (SMD 5.87; 95% CI: –8.37; 20.11) compared with dobutamine. Treatment with levosimendan also resulted in significant reductions in BNP (SMD –1.87; 95% CI: –2.45; −1.2) and serum lactate levels (SMD –1.63; 95% CI: –3.13; −0.12). However, TSA indicated that the current evidence is insufficient to definitively confirm or exclude effects on in-hospital and 28-day mortality. **Conclusions:** Levosimendan may improve hemodynamics, tissue perfusion, and biomarkers, and may reduce in-hospital mortality and ICU length of stay in patients with SICM compared with dobutamine. However, TSA highlights the need for further studies to inform clinical practice and optimize inotrope selection.

## 1. Introduction

Sepsis is a common and potentially life-threatening condition that affects millions of people each year, with mortality rates ranging from 40% to 60% [1]. Sepsis and septic shock are considered independent risk factors for mortality in critically ill patients [2]. Numerous interventions have been proposed to reduce mortality rates in this complex condition. The Surviving Sepsis Campaign (SSC) was launched in 2002 as a collaborative initiative by the European Society of Intensive Care Medicine (ESICM), the International Sepsis Forum (ISF), and the Society of Critical Care Medicine (SCCM), with the goal of developing evidence-based guidelines to reduce the high mortality associated with this prevalent condition [3]. The implementation of care bundles, incorporating measurable actions such as early administration of intravenous (IV) fluids, broad-spectrum antimicrobial therapy, and vasopressor support, has since become standard practice [1,4].

The most significant global reduction in septic shock mortality was reported between 2009 and 2011 [2]. Many ongoing trials are currently focused on modulating the immune response to reduce mortality [5]. However, even when early disease mechanisms are addressed through immune modulation and hemodynamic stabilization, clinicians must recognize that most sepsis and septic shock cases are diagnosed after the systemic inflammatory response syndrome (SIRS) has already developed [6].

Septic-induced cardiomyopathy (SICM) is a common hemodynamic disturbance in septic shock. Myocardial dysfunction, resulting from various inflammatory pathways, leads to ventricular dilatation [7], increased ventricular compliance with normal to low filling pressures, reduced left ventricular ejection fraction (EF), and diminished response to both fluid resuscitation and catecholamines. This results in persistent hypoperfusion and organ failure [8]. Current recommendations include the use of IV fluids and inotropic therapy guided by different hemodynamic monitoring techniques [9]. Although there is no universally accepted definition of SICM, it is generally characterized by impaired cardiac function, as assessed by echocardiography, pulmonary artery catheterization, transpulmonary thermodilution, or cardiac biomarkers [10].

Regarding inotropic agents, several studies have evaluated the effectiveness of different molecules for treating SICM [11]. The SSC currently recommends dobutamine, while levosimendan is not endorsed for routine use [1]. A meta-analysis by Bhattacharjee et al. found no significant difference in mortality between patients treated with dobutamine versus levosimendan for SICM (OR 0.80, 95% CI: 0.48; 1.33) [12]. Moreover, levosimendan is associated with higher healthcare costs, which may reduce its adoption in clinical practice. Nonetheless, levosimendan’s pleiotropic effects on the myocardium and microcirculation [13] warrant further evaluation. This study aims to review new clinical evidence comparing different inotropic agents, in line with the SSC’s original goal of guiding sepsis management through evidence-based interventions. Therefore, we conducted a meta-analysis of randomized controlled trials (RCTs) comparing dobutamine and levosimendan as inotropic agents for treating SICM—defined by documented cardiac dysfunction—to assess whether levosimendan confers benefits over dobutamine in this patient population.

## 2. Materials and Methods

### 2.1. Study Protocol

The review protocol was registered in the International Prospective Register of Systematic Reviews (PROSPERO) under registration number CRD42024605325. This systematic review and meta-analysis were conducted and reported according to the Preferred Reporting Items for Systematic Reviews and Meta-Analyses (PRISMA) guidelines.

### 2.2. Study Selection

We conducted a systematic search of MEDLINE, EMBASE, and the Cochrane Library (Cochrane Database of Systematic Reviews and CENTRAL) through October 2024. Boolean operators were used with the following MeSH terms: “Shock, Septic,” “Sepsis,” “Simendan,” “Mortality,” “Myocardium,” and “Heart.” Filters were applied to include only clinical trials. Titles and abstracts were independently screened using Rayyan software, and full texts were retrieved for relevant studies.

We included RCTs comparing levosimendan and dobutamine in patients with septic shock who met the following criteria: (1) required vasopressor support and/or had evidence of hypoperfusion (lactate > 2 mmol/L), and (2) had clinical or biochemical evidence of myocardial dysfunction. Studies comparing levosimendan with therapies other than dobutamine, and articles not in English, were excluded. Although the LeoPARDS trial by Gordon et al. [14]—the largest RCT of levosimendan in sepsis—did not meet our inclusion criteria (only 52 patients had documented myocardial dysfunction), we performed a secondary analysis that included 24 patients in the placebo group who received dobutamine, despite this not fully meeting our predefined criteria.

Primary outcomes included ICU mortality, hospital discharge mortality, and 28-day mortality. Secondary outcomes included hemodynamic parameters (mean arterial pressure [MAP], left ventricular ejection fraction [LVEF], and cardiac index [CI]) before and after intervention, cardiac biomarkers (BNP, TnI), serum lactate, hospital length of stay, and duration of mechanical ventilation. Relevant review articles were referenced for discussion. The Supplementary Materials present outcome results that include the LeoPARDS subgroup receiving dobutamine.

### 2.3. Data Extraction

Two investigators (LR and SP) independently extracted data using standardized forms. Discrepancies were resolved through discussion with two additional reviewers (DR and GD). Extracted data included author, year of publication, study design, sample size, baseline characteristics, and all reported outcomes.

### 2.4. Statistical Analysis

The meta-analysis was conducted using R Studio (v4.3.1). A random-effects model was applied. Odds ratios (ORs) were used for categorical outcomes and standardized mean differences (SMDs) for continuous outcomes. Binary outcome weights were calculated using the Mantel-Haenszel method, and pooled mean differences were estimated using the Hedges method. Heterogeneity was assessed using the Chi-square test (*p* > 0.1 indicating homogeneity) and Higgins’ I^2^ statistic (I^2^ > 50% indicating substantial heterogeneity). Sensitivity analyses were performed for outcomes with data from ≥5 studies. The influence of individual studies and their contributions to heterogeneity in in-hospital and 28-day mortality were evaluated graphically.

### 2.5. Risk of Bias in Individual Studies

Risk of bias was assessed using the Cochrane Risk of Bias (ROB) tool. Two investigators (LR and SP) independently evaluated each study. Discrepancies were resolved through discussion to achieve consensus.

### 2.6. Publication Bias

When ≥5 studies were available for an outcome, publication bias was assessed using funnel plots and Egger’s linear regression test.

### 2.7. Subgroup Analysis and TSA

The robustness of the primary outcomes (in-hospital and 28-day mortality) was further evaluated using trial sequential analysis (TSA) in R Studio (v4.3.1) with the RTSA package. ORs from the meta-analysis were used to assess cumulative evidence against trial sequential monitoring boundaries. A random-effects model was applied with 80% power and 5% significance.

## 3. Results

### 3.1. Study Selection and Characteristics of the Included Trial

**Figure 1:**
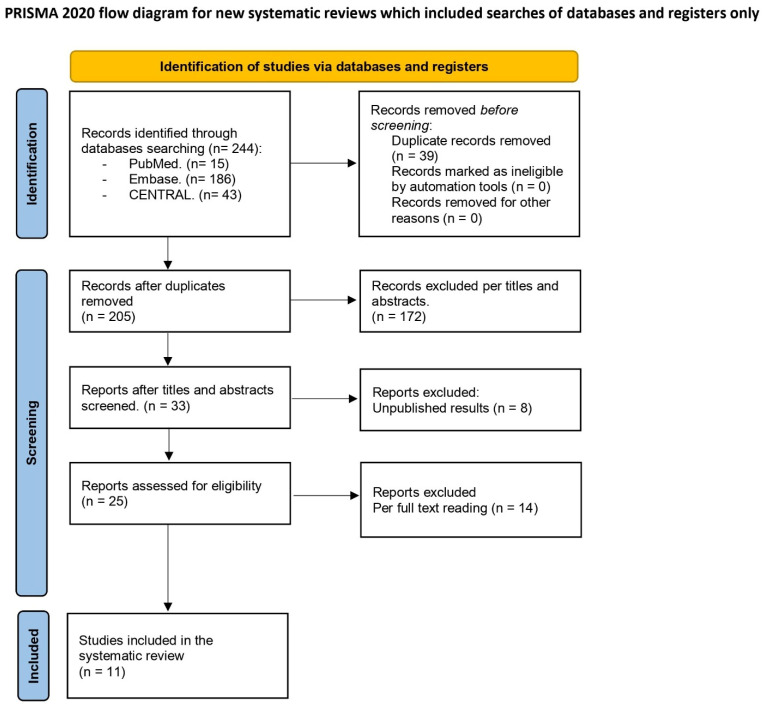
PRISMA flowchart.

The electronic search identified a total of 244 studies. After removing 39 duplicates, 172 studies were excluded based on titles and abstracts. Additionally, eight studies registered in CENTRAL were excluded due to the unavailability of results. Twenty-five full-text articles were reviewed for eligibility, with fourteen excluded for not meeting the inclusion criteria. Ultimately, 11 studies were included in the final analysis (references [15,16,17,18,19,20,21,22,23,24,25]). The screening and selection process is detailed in Figure 1, and Table 1 provides a summary of the main characteristics and findings of the included studies.

### 3.2. ROB in the Studies

**Figure 2:**
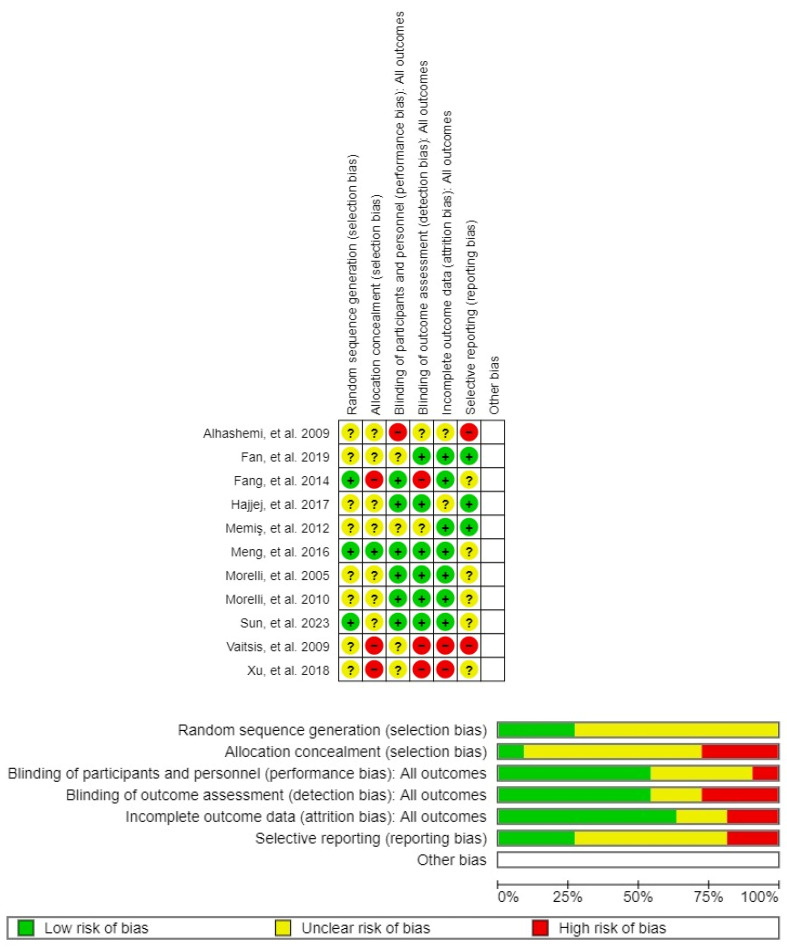
Distribution of the risk of bias assessment [15,16,17,18,19,20,21,22,23,24,25].

The Cochrane RoB2 tool was used to assess the ROB, revealing that all 11 included studies had at least one domain with an unclear ROB. The study by Meng et al. [16] was identified as having the lowest ROB (Figure 2).

### 3.3. Mortality Analysis

**Figure 3:**
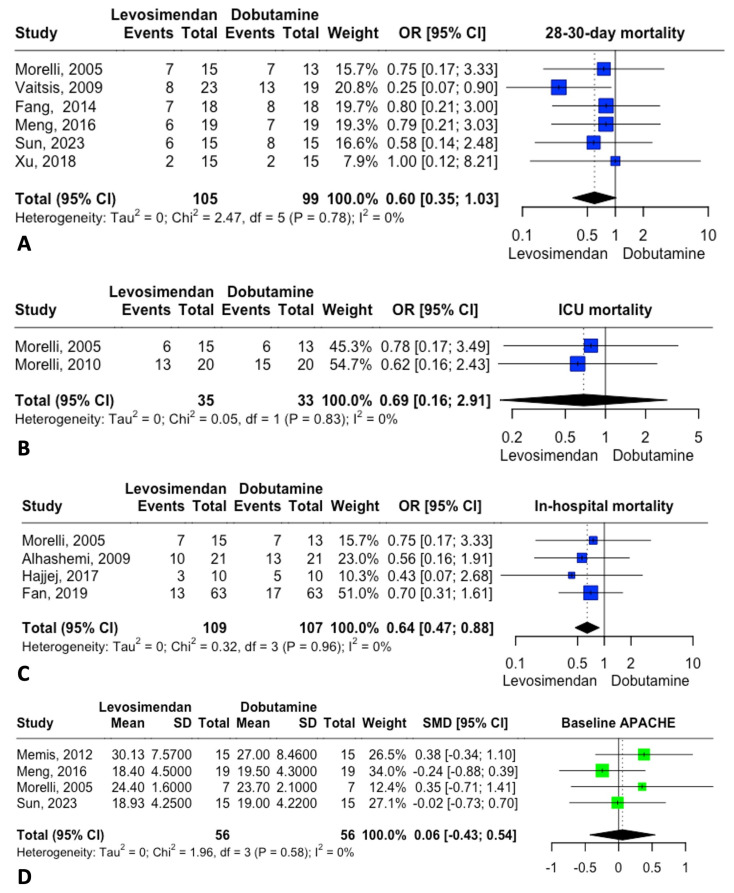
Forrest plot for the main outcomes. (**A**) 28–30-day mortality. (**B**) Intensive care unit (ICU) Mortality. (**C**) In-hospital mortality. (**D**) Baseline APACHE.

**Figure 4:**
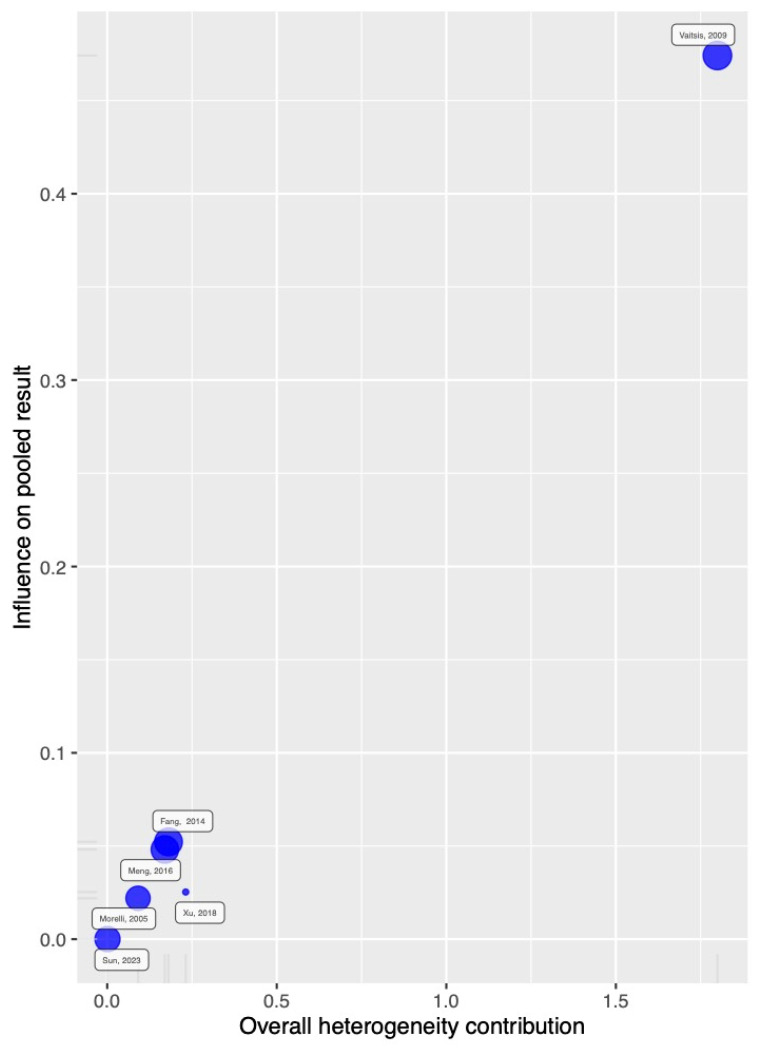
Overall heterogeneity contribution and influence on the result for 28–30-day mortality.

The primary outcome was 28–30-day mortality. Six studies reported this outcome, showing a trend toward reduced mortality without statistical significance. Figure 3 displays an OR of 0.60 [95% CI: 0.35; 1.03]. A sensitivity analysis suggested that statistical significance might be achieved if the study by Vaitsis [21] were omitted (Figure 4). No publication bias was observed for this outcome.

In-hospital mortality was assessed in four studies. The analysis yielded an OR of 0.64 [95% CI: 0.47; 0.88], suggesting a potential beneficial effect of levosimendan in reducing in-hospital mortality (Figure 3).

Baseline characteristics were deemed important for both groups. The APACHE II score, reported in four studies, indicated no significant baseline differences in patient status at the time of intervention (Figure 3).

**Figure 5:**
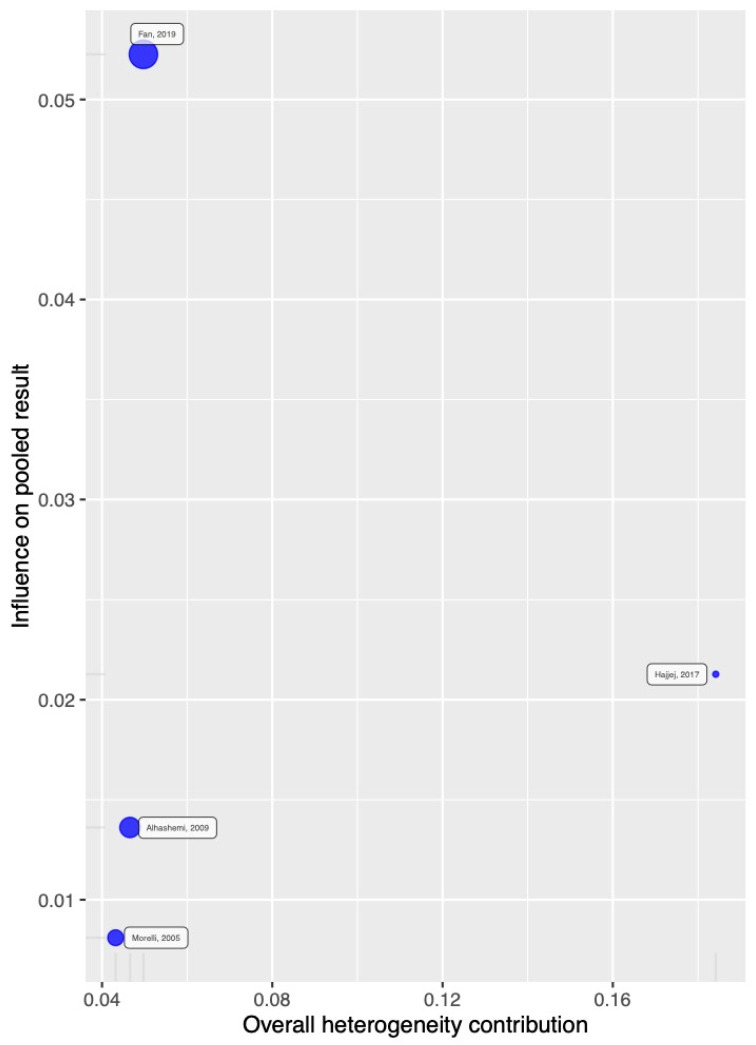
Overall heterogeneity contribution and influence on the result for In-Hospital mortality.

The influence and heterogeneity contribution from each study to 28–30-day mortality are shown in Figure 4, and those for in-hospital mortality are shown in Figure 5.

#### Secondary Analysis with LeoPARDS

The analysis of 28–30-day mortality across seven studies, including the LeoPARDS trial, continued to show a trend toward reduced mortality without statistical significance (OR 0.84 [95% CI: 0.3; 0.32]; Supplementary Figure S1). Heterogeneity in the meta-analysis increased, with an I^2^ of 42%.

In-hospital mortality was assessed in five studies, including the LeoPARDS subgroup. This also suggested a potential benefit, though without statistical significance (OR 0.98 [95% CI: 0.20; 4.75]). However, group imbalance was evident due to the small sample size in the dobutamine group (n = 24; Supplementary Figure S2).

### 3.4. Biomarkers

**Figure 6:**
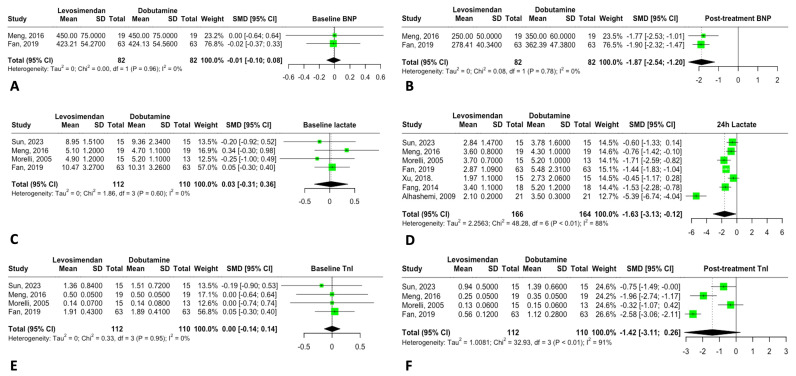
Forrest plot for biomarkers. (**A**) Baseline BNP. (**B**) Post-treatment BNP. (**C**) Baseline lactate. (**D**) 24h lactate. (**E**) Baseline Troponin I (TnI). (**F**) Post-treatment TnI.

**Figure 7:**
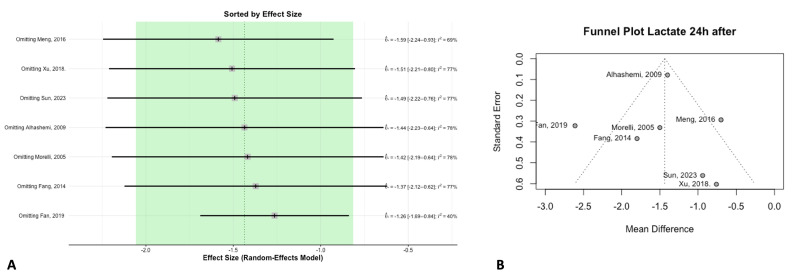
Lactate levels. (**A**) Sensitivity analysis for lactate levels. (**B**) Publication bias assessment by funnel plot.

The biomarkers analyzed—BNP, lactate, and troponin—showed no significant baseline differences between the groups. However, BNP levels showed a significant reduction following levosimendan treatment, suggesting improved cardiac filling pressures. Lactate levels also decreased 24 h after levosimendan administration, indicating improved systemic tissue perfusion (Figure 6). These findings remained consistent after sensitivity analysis (Figure 7A), with no evidence of publication bias (Figure 7B).

### 3.5. Hemodynamic Parameters and ICU Stay

Baseline LVEF showed no significant differences between the groups. However, after 24 h of treatment with levosimendan, a significant improvement in LVEF was observed compared to dobutamine (MD: 5.49 [95% CI: 3.61; 7.37]), suggesting a positive effect on cardiac function.

For MAP, no significant baseline differences were noted, and 24 h post-treatment, levosimendan did not increase MAP compared to dobutamine (MD: 5.87 [95% CI: –8.37; 20.11]); however, there was high heterogeneity (I^2^ = 97%).

**Figure 8:**
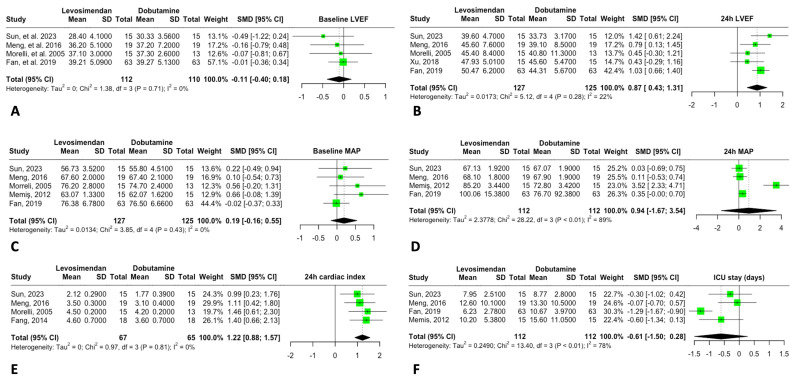
Forrest plot for Hemodynamics. (**A**) Baseline Left Ventricular Ejection Fraction (LVEF). (**B**) 24h LVEF. (**C**) Baseline Mean arterial pressure (MAP). (**D**) 24h MAP. (**E**) 24h Cardiac index. (**F**) ICU stay (days).

ICU length of stay was reported in four studies, with a shorter stay in the levosimendan group compared to the dobutamine group (MD: –2.84 [95% CI: –6.43; 0.74]). This reduction may reflect overall clinical improvement with levosimendan, though interpretation should be cautious due to variability across studies (Figure 8).

### 3.6. TSA

**Figure 9:**
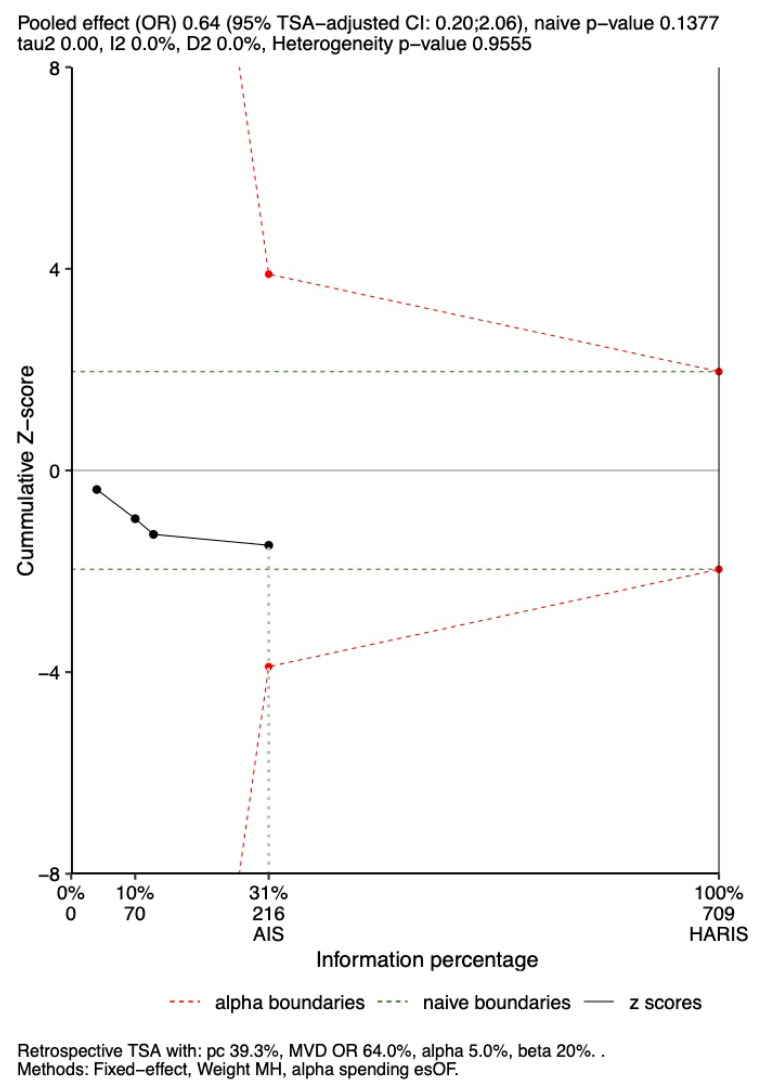
Trial sequential analysis for In-Hospital Mortality. AIS: achieved information size; HARIS: heterogeneity-adjusted required information size for a non-sequential meta-analysis; MVD: mean value difference; DL_HKSJ: DerSimonian–Laird with Hartung–Knapp–Sidik–Jonkman adjustment; esOF: Lan and DeMets version of O’Brien–Fleming boundaries; TSA: trial sequential analysis.

**Figure 10:**
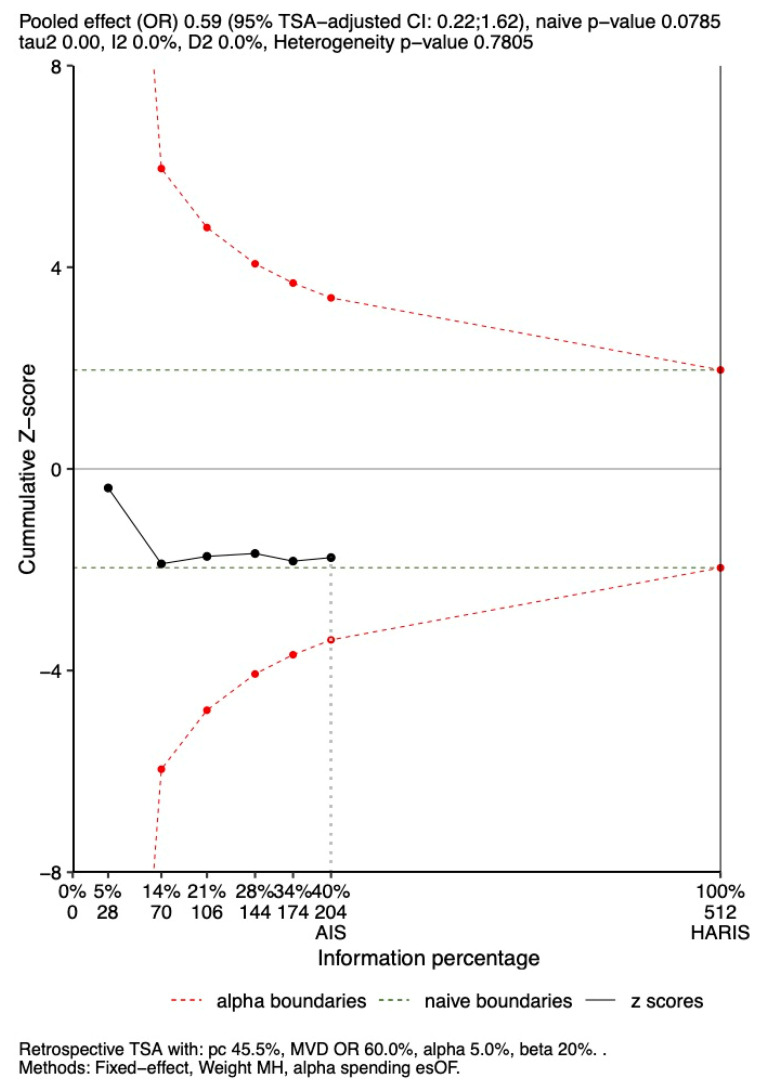
Trial sequential analysis for 20–30-day Mortality. AIS: achieved information size; HARIS: heterogeneity-adjusted required information size for a non-sequential meta-analysis; MVD: mean value difference; DL_HKSJ: DerSimonian–Laird with Hartung–Knapp–Sidik–Jonkman adjustment; esOF: Lan and DeMets version of O’Brien–Fleming boundaries; TSA: trial sequential analysis.

TSA was performed to assess the robustness of findings for in-hospital and 28-day mortality and is shown in Figure 9 and Figure 10. TSA indicated that the results were not robust for either outcome. The cumulative Z-scores remained within the boundaries for non-significance, using the ORs of 0.64 and 0.59, respectively, derived from the primary meta-analysis.

#### 3.6.1. In-Hospital Mortality

The observed OR of 0.64 for in-hospital mortality fell within the TSA-adjusted boundaries (OR 0.2–2.06; *p* = 0.13). However, the analysis suggests that the results are not robust, as the cumulative Z-score did not cross the significance threshold. Additionally, the analyzed sample represents only 31% of the 709 participants needed to achieve 90% power (Figure 9).

#### 3.6.2. 28-Day Mortality

The OR of 0.60 for 28-day mortality also fell within the TSA-adjusted boundaries (OR 0.2–1.62; *p* = 0.07). Similarly, the cumulative Z-score did not cross the significance threshold. The analyzed sample represents only 40% of the 512 participants needed to achieve 90% statistical power (Figure 10).

## 4. Discussion

This study compared 11 trials evaluating levosimendan versus dobutamine in patients with septic shock and suspected myocardial dysfunction based on hemodynamic parameters, biomarkers, and lack of response to standard medical therapy. SICM was defined in each study by either a reduction in LVEF or the requirement for inotropic therapy to achieve hemodynamic goals.

Currently, the management of septic shock is considered standardized, as guided by the SSC recommendations through the application of care bundles [1,26]. The first 24 h of resuscitation focus on salvage and optimization, guided by MAP, cardiac output monitoring, capillary refill time, lactate levels, venous oxygen saturation (SvO_2_), and the arterio-venous carbon dioxide difference [27]. The use of inotropes, in addition to IV fluids and vasopressors, is a cornerstone of management during the optimization and stabilization phases, particularly when a cardiogenic component is suspected. However, some patients may continue to exhibit high cardiac output even in later phases of resuscitation and may not benefit from inotropic therapy [28].

This meta-analysis showed that levosimendan significantly reduces in-hospital mortality (OR 0.64, 95% CI: 0.47; 0.88) and also shows a trend toward lower 30-day mortality. This trend has also been identified by Guan et al., who reported reduced mortality with levosimendan (RR 0.75, 95% CI: 0.60; 0.95) [29]. Multiple studies have demonstrated the beneficial effects of levosimendan in various shock states, mainly cardiogenic [30,31] and vasoplegic [14] shock, as well as its pleiotropic effects, including facilitating weaning from mechanical ventilation [32,33] and mechanical circulatory support [34,35]. The observed mortality reduction with levosimendan may be attributed to improved hemodynamics and resolution of organ dysfunction, promoting optimal organ perfusion.

This meta-analysis included only patients with defined SICM, diagnosed using either hemodynamic parameters or cardiac biomarkers, and employed TSA to assess whether the sample size was adequate to support the hypothesis. It did not include the LeoPARDS trial, one of the largest studies investigating levosimendan in sepsis. Upon examining its design, several methodological concerns may explain why our analysis trended toward a favorable outcome for levosimendan in SICM. First, in LeoPARDS, fewer than 10% of patients in the placebo group received dobutamine (24 patients); second, only 52 patients were documented with SICM criteria (low cardiac index); and third, only one patient in the dobutamine group died, likely due to the small sample size. Our secondary exploratory analysis (available in the Supplementary Materials) does not exclude the potential benefit of levosimendan in SICM.

In this meta-analysis, a significant improvement in LV function was observed in terms of CI and LVEF, while no significant difference in MAP was found between the two groups 24 h after treatment, suggesting safety. These differences can be attributed to the drugs’ mechanisms of action: levosimendan acts as a calcium sensitizer, whereas dobutamine relies on catecholamine stimulation. In septic patients, levosimendan’s cAMP-independent effects may reduce oxygen demand and the risk of arrhythmias, potentially minimizing catecholamine resistance [36].

Cardiac biomarkers are often elevated in sepsis, but high levels are not solely indicative of sepsis-induced cardiac dysfunction. Elevations may result from neurohormonal activation, volume resuscitation, sepsis-induced ventricular dilation, or stimulation by lipopolysaccharides and pro-inflammatory cytokines [37]. Nonetheless, their trends may aid in guiding inotropic therapy decisions [27]. Our analysis found that levosimendan was more effective in reducing BNP compared to dobutamine, possibly due to its effects on end-diastolic wall tension. Although levosimendan may prevent troponin degradation without affecting intracellular calcium levels, no significant differences in troponin levels were observed between the groups [38]. Some studies in cardiogenic shock have similarly shown no significant changes in cardiac biomarkers [39,40].

No differences in hypotensive events were observed between levosimendan and dobutamine, suggesting that levosimendan is safe, even considering its vasodilatory properties. As a vasodilator, levosimendan improves microcirculatory flow and oxygen delivery by enhancing hemodynamic coherence [13,41,42]. This is crucial, as microcirculatory disturbances may persist in septic shock even when macro-hemodynamic parameters appear normal [43].

Levosimendan’s anti-inflammatory effects have been studied in cecal ligation and puncture (CLP) and lipopolysaccharide (LPS) models [44]. In our analysis, lactate levels were significantly reduced with levosimendan use, indicating improved tissue perfusion, consistent with previous studies [45,46,47]. Since lactate reduction is a therapeutic goal [26], this may serve as a marker of favorable prognosis in septic shock [48]. Beyond its inotropic action, levosimendan’s immunomodulatory and anti-inflammatory properties may contribute to improved cellular perfusion and lower lactate levels [49]. However, limited evidence supports these anti-inflammatory effects. In a subgroup analysis of the LeoPARDS trial [14], Antcliffe et al. reported only a modest trend toward reduced inflammatory biomarkers (e.g., CCL2, IL-6, IL-8, IL-10, and sTNFr1) with levosimendan use [50].

TSA, a frequentist method that applies predefined type I and type II error thresholds, evaluates whether meta-analysis results are sufficiently robust to remain unaffected by future studies. TSA clarifies whether the required sample size has been reached to draw definitive conclusions [51]. In our case, TSA indicates a zone of uncertainty, emphasizing the need for further head-to-head studies comparing levosimendan and dobutamine.

Despite non-conclusive data on the duration of mechanical ventilation, levosimendan remains a promising option, as it may enhance diaphragmatic function [52]. This benefit is still under investigation and may be more pronounced in patients requiring prolonged mechanical ventilation. Additionally, while 28-day mortality did not show a statistically significant difference, in-hospital mortality did. TSA demonstrated that the ORs for both outcomes remain in the significant region; however, the total required sample size has not yet been achieved.

This updated meta-analysis, incorporating the latest evidence and a rigorous TSA methodology, supports consideration of levosimendan as a first-line inotropic agent in SICM—defined by objective evidence of cardiac dysfunction. Nevertheless, due to high heterogeneity and small sample sizes in most included studies, further research is necessary before formal recommendations can be integrated into SSC guidelines and care bundles aimed at reducing mortality in sepsis and septic shock.

## 5. Conclusions

The use of levosimendan in patients with SICM may offer advantages over dobutamine, including potential improvements in hemodynamics, perfusion markers, and cardiac biomarkers. It may also contribute to reductions in in-hospital hospital mortality and ICU length of stay. However, TSA indicates that further studies are required to guide clinical practice and optimize treatment selection.

Large-scale RCTs are needed to confirm levosimendan’s benefits and clarify its role in the management of septic shock with myocardial dysfunction.

## 6. Limitations

This meta-analysis has several limitations. First, incomplete reporting of baseline characteristics in some studies may introduce confounding factors and hinder comparability across patient populations. Additionally, significant heterogeneity—particularly in outcomes such as MAP—limits the generalizability of the findings. Small sample sizes in several studies may also reduce statistical power and increase variability in effect estimates.

Variation in the definition of myocardial dysfunction and inconsistencies in treatment protocols across studies may further affect the pooled outcomes. Moreover, this meta-analysis did not include studies comparing levosimendan with placebo; therefore, findings should be interpreted strictly in the context of head-to-head comparisons between levosimendan and dobutamine. Although the overall effect appears to favor levosimendan, TSA indicates that a larger sample size is needed to draw definitive conclusions.

Potential anti-inflammatory effects and reductions in cardiac biomarkers should be interpreted with caution, as the supporting evidence remains inconclusive and varies across different patient groups.

Finally, publication bias cannot be ruled out, as studies with negative or null findings may be under-reported.

## Figures and Tables

**Table 1:** Summary of the included trials [15,16,17,18,19,20,21,22,23,24,25].

Study	Year	Levosimendan Group	Control Group	Levosimendan Dose (IV)	Dobutamine Dose (IV)	Inclusion Criteria	Outcomes
Sun, et al. [15]	2023	15	15	0.2 μg/kg/min for 24 h	5 μg/kg/min for 24 h.	Patients within the first 48 h from sepsis onset, with MAP maintained at 65 mmHg with norepinephrine, who presented LVEF ≤ 35% after fluid resuscitation and increase in myocardial injury markers after fluid resuscitation and vasoactive drug treatment.	Mortality 28-day. Hemodynamics. NE dose. Lactate, cTnI, NTproBNP, and PCR levels. Length of mechanical ventilation. ICU stay (days) ICU costs.
Meng, et al. [16]	2016	19	19	0.2 μg/kg/min for 24 h.	5 μg/kg/min for 24 h.	Patients with septic shock and established normovolemia using norepinephrine to maintain MAP of at least 65 mmHg, with LVEF ≤ 45% after fluid resuscitation and vasopressor therapy.	Hemodynamics. HFABP, TNI, BNP, and Lactate levels. Mortality 28 days. ICU stay. Hospital stay. Length of mechanical ventilation. NE dose (baseline and 24 h).
Morelli, et al. [17]	2010	20	30	0.2 μg/kg/min for 24 h.	5 μg/kg/min for 24 h.	Patients within the first 24 h from the onset of septic shock after having established normovolemia and an MAP of at least 65 mmHg using norepinephrine.	Hemodynamics. Microcirculatory flow variables. Lactate. NE dose (baseline and 24 h). Mortality (ICU). ICU stay.
Morelli, et al. [18]	2005	15	13	0.2 μg/kg/min for 24 h.	5 μg/kg/min for 24 h.	Patients within the first 24 h from the onset of septic shock after having established normovolemia, norepinephrine, and dobutamine given to maintain MAP of at least 65 mmHg while maintaining Hb > 7 g/dL.	Hemodynamics. Gastric perfusion. Lactate and TnI levels. Mortality (in-hospital, ICU, 30-day).
Fan, et al. [19]	2019	63	63	Bolus 6–12 μg/kg, continued at 0.1 μg/kg/min for 24 h.	5 μg/kg/min for 3 days.	Patients with septic shock managed under early goal-directed therapy, needing norepinephrine for blood pressure maintenance.	Cardiac function parameters. Lactate, BNP, PCT, and cTnI concentrations. APACHE II score. NE total dose. O_2_ inhalation time of mechanical ventilation. In-hospital mortality. ICU stay.
Memiş, et al. [20]	2012	15	15	0.1 μg/kg/min for 24 h.	10 μg/kg/min for 24 h.	Critically ill patients who met at least two of the criteria of septic shock, as defined by CHEST, with MAP of ≤65 mmHg despite dopamine infusion.	Liver function. Hemodynamic variables. ICU stay.
Vaitsis, et al. [21]	2009	23	19	0.1 μg/kg/min for 24 h	5–10 μg/kg/min for 24 h.	Patients with sepsis and severe cardiac dysfunction (CI ≤ 2.2, LVEF ≤ 35%).	Mortality at 7 and 30 days.
Alhashemi, et al. [22]	2009	21	21	0.05–0.2 μg/kg/min for 24 h.	5–20 μg/kg/min for 7 days maximum.	Patients with severe sepsis or septic shock managed with norepinephrine infusion titrated to an MAP of at least 65 mmHg.	ICU mortality, first-day serum lactate.
Hajjej, et al. [23]	2017	10	10	0.2 μg/kg/min for 24 h.	5 μg/kg/min for 72 h.	Patients with septic shock requiring norepinephrine to maintain an MAP of at least 65 mmHg despite appropriate volume resuscitation.	Systemic hemodynamics. Global oxygen transport. Acid-base homeostasis. Muscle microdialysis variables.
Xu, et al. [24]	2018	15	15	0.2 μg/kg/min for 24 h.	5 μg/kg/min for 24 h.	Elderly patients with sepsis with LVEF ≤ 50% after fluid resuscitation.	Cardiac function parameters. Lactate levels. Length of mechanical ventilation. ICU stay. Mortality 28-day.
Fang, et al. [25]	2014	18	18	Dobutamine + 0.2 μg/kg/min for 24 h.	5 μg/kg/min for 48 h.	Patients with septic shock with LVEF ≤ 45% after fluid resuscitation.	Hemodynamics and cardiac function. Lactate levels. 24-h urinary output. NE total dose. Mortality (ICU and 28-day).

## Data Availability

Data is available and will be shared upon request to the corresponding author.

## Supplementary Materials

The following supporting information can be downloaded at: https://www.mdpi.com/article/10.3390/jcm14155496/s1, Figure S1: Forrest plot for 28–30-day mortality including 24 studied patients with dobutamine in LeoPARDS trial, Figure S2: Forrest plot for In-Hospital mortality including 24 studied patients with dobutamine in LeoPARDS trial.

## Abbreviations

SICM	Septic-induced cardiomyopathy
TSA	Trial sequential analysis
OR	Odds ratio
CI	Confidence interval
SMD	Standardized mean difference
MAP	Mean arterial pressure
LVEF	Left ventricular ejection fraction
BNP	Brain natriuretic peptide
ICU	Intensive care unit
APACHE II	Acute Physiology and Chronic Health Evaluation
TnI	Troponin I
