# Supplementary material for: Levosimendan vs. Dobutamine in Patients with Septic Shock: A Systematic Review and Meta-Analysis with Trial Sequential Analysis
Source: J Clin Med. 2025 Aug 5;14(15):5496. doi: 10.3390/jcm14155496 (PMC12347308; doi:10.3390/jcm14155496)
Supplement: Supplementary file 1 [file jcm-14-05496-s001.zip › jcm-3663135-supplementary.pdf]

Supplementary Appendix

Study	Levosimendan Events	Levosimendan Total	Dobutamine Events	Dobutamine Total	Weight	OR [95% CI]
Morelli, 2005	7	15	7	13	14.8%	0.75 [0.17; 3.33]
Vaitsis, 2009	8	23	13	19	16.9%	0.25 [0.07; 0.90]
Fang, 2014	7	18	8	18	16.5%	0.80 [0.21; 3.00]
Meng, 2016	6	19	7	19	16.3%	0.79 [0.21; 3.03]
Sun, 2023	6	15	8	15	15.2%	0.58 [0.14; 2.48]
Xu, 2018	2	15	2	15	9.9%	1.00 [0.12; 8.21]
Gordon, 2016	90	259	1	24	10.4%	12.25 [1.63; 92.18]
**Total (95% CI)**		**364**		**123**	**100.0%**	**0.84 [0.30; 2.31]**

Heterogeneity: $Tau^2 = 0.5834$; $Chi^2 = 10.41$, df = 6 (P = 0.11); $I^2 = 42\%$

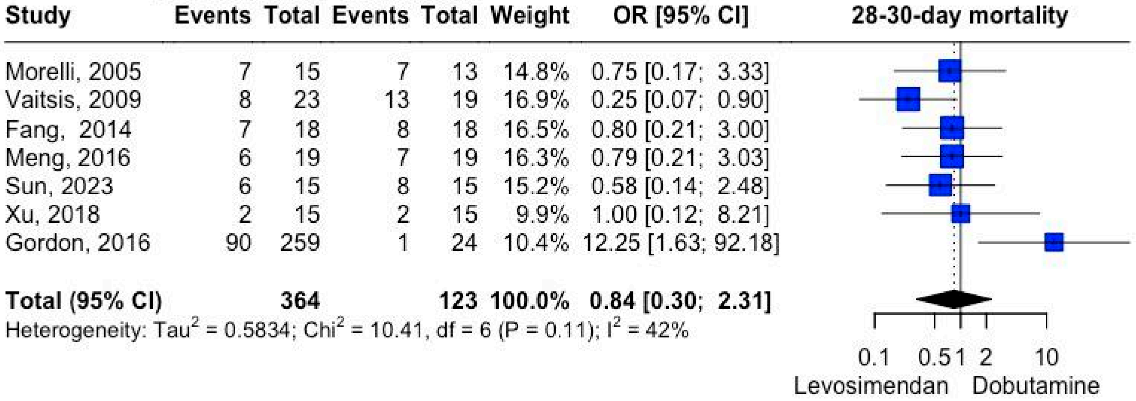

Figure 1. Outcome: 28-30-day mortality including 24 studied patients with dobutamine in LeoPARDS trial.

Study	Levosimendan Events	Levosimendan Total	Dobutamine Events	Dobutamine Total	Weight	OR [95% CI]
Morelli, 2005	7	15	7	13	19.7%	0.75 [0.17; 3.33]
Alhashemi, 2009	10	21	13	21	22.2%	0.56 [0.16; 1.91]
Hajjej, 2017	3	10	5	10	16.7%	0.43 [0.07; 2.68]
Fan, 2019	13	63	17	63	26.1%	0.70 [0.31; 1.61]
Gordon, 2016	97	259	1	24	15.2%	13.77 [1.83; 103.59]
**Total (95% CI)**		**368**		**131**	**100.0%**	**0.98 [0.20; 4.75]**

Heterogeneity: $Tau^2 = 1.0583$; $Chi^2 = 8.51$, df = 4 (P = 0.07); $I^2 = 53\%$

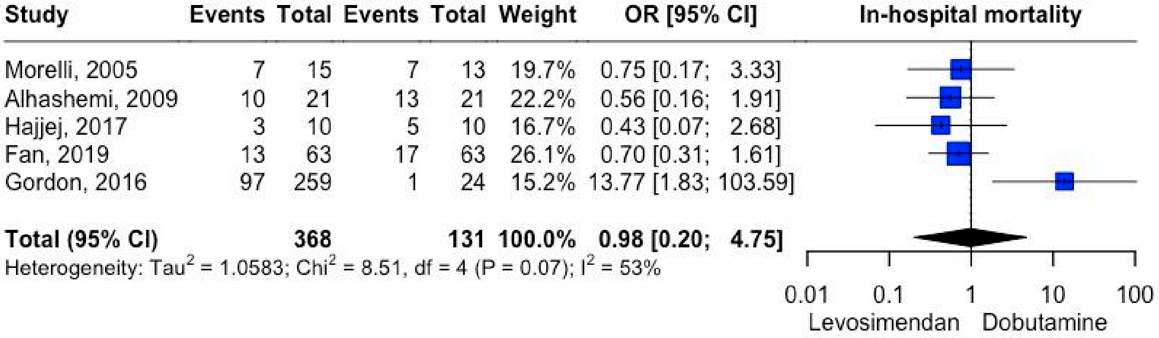

Figure 2. Outcome: In-Hospital mortality including 24 studied patients with dobutamine in LeoPARDS trial.
